# Magnetic reversal dynamics of a quantum system on a picosecond timescale

**DOI:** 10.3762/bjnano.6.199

**Published:** 2015-09-28

**Authors:** Nikolay V Klenov, Alexey V Kuznetsov, Igor I Soloviev, Sergey V Bakurskiy, Olga V Tikhonova

**Affiliations:** 1 Lomonosov Moscow State University Physics Department, Moscow 119991, Russia; 2 Lomonosov Moscow State University Skobeltsyn Institute of Nuclear Physics, Moscow 119991, Russia; 3 Lukin Scientific Research Institute of Physical Problems, Zelenograd, Moscow 124460, Russia; 4 Moscow Institute of Physics and Technology, State University, Dolgoprudniy, Moscow Region, Russia

**Keywords:** atomic-based qubits, magnetization reversal, quantum state control, RSFQ, superconducting qubits

## Abstract

We present our approach for a consistent, fully quantum mechanical description of the magnetization reversal process in natural and artificial atomic systems by means of short magnetic pulses. In terms of the simplest model of a two-level system with a magnetic moment, we analyze the possibility of a fast magnetization reversal on the picosecond timescale induced by oscillating or short unipolar magnetic pulses. We demonstrate the possibility of selective magnetization reversal of a superconducting flux qubit using a single flux quantum-based pulse and suggest a promising, rapid Λ-scheme for resonant implementation of this process. In addition, the magnetization reversal treatment is fulfilled within the framework of the macroscopic theory of the magnetic moment, which allows for the comparison and explanation of the quantum and classical behavior.

## Introduction

The study of magnetic moment dynamics in atomic systems (including Rydberg atoms) is one of the simplest ways to monitor the evolution of quantum states. Two-level quantum systems with a well-defined magnetic moment of two magnetic basis states, 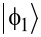 and 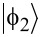, continue to attract considerable attention in the context of the development of modern systems for information processing and storage. The significantly suppressed transition probability between the magnetic states (in comparison to electric dipole transitions) makes it difficult to achieve subnanosecond characteristic times for logical operations (e.g., initialization processes, i.e., preparation of 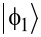 and 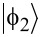 superposition with given amplitudes and “write” operations in magnetic memory cells). In this article we investigate ways to control the quantum dynamics of a magnetic system on an extremely short timescale and suggest promising physical implementations based on the simple two-level model.

The superconducting flux qubit, a leading candidate for scalable quantum information processing in the field of solid state devices, demonstrates a macroscopically large value of the magnetic moment in quantum basis states. To date, mesoscopic artificial atoms based on a superconducting loop with Josephson junctions have been successfully created. The control technique developed for these effectively two-level pseudo-atomic systems allows for the implementation of single- and multi-qubit operations. Moreover, the observation of effects referred to as “quantum optics on a chip” is possible, leading to a probe on the border between classical and quantum mechanics [1–15].

The possible gate and measurement rates estimated in relation to the decoherence processes for these qubits are within the reach of the threshold for fault-tolerant quantum computing. In order to accomplish the last step, the time requirements for basic operations and initialization must be reduced. The direction for integration of a multiqubit quantum processor with well-known, cold, single flux, quantum-based (SFQ) digital circuitry for both control and measurement procedures has already been identified [16–24]. Quasi-solitary current and voltage waves (called fluxons) are obtained in strongly nonlinear, one-dimensional Josephson media (Josephson transition line). Fluxons carry magnetic flux quanta, Φ_0_, in RSFQ circuits and serve as magnetic field sources for fast qubit control. However, the important features of the flux qubit dynamics under an external magnetic field have still not been studied in detail, especially regarding the initial state preparation and logical operations performed with the RSFQ-bit circuits. In this context, the optimization of the magnetization reversal on the shortest timescale possible is highly relevant.

**Figure 1:**
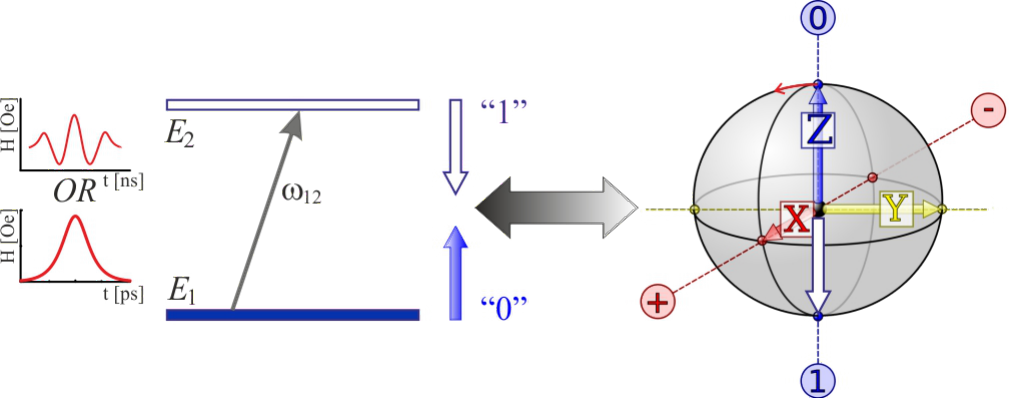
Magnetization reversal of a two-level system is shown as a transition between the states “0” and “1” for certain values of the magnetic moment (these states correspond to the energy levels of *E*_1_ and *E*_2_, respectively) by either a unipolar or oscillating magnetic field. The given transition is somewhat analogous to the rotation of the Bloch vector or to the precession of the magnetic moment under the influence of a magnetic field perpendicular to the plane of precession.

In this work, we investigate the dynamics of a magnetic system under an external magnetic field and focus on the achievement of a short magnetization reversal duration down to the picosecond timescale. The typical operation for the superconducting flux qubits can be described as follows: the qubit is prepared in one of the magnetic basis eigenstates and then driven to the degeneracy point by an external flux applied to its loop. Therefore, the minimum time required to flip the qubit in this manner is half of the period for coherent oscillations between qubit states (50 ps if we assume the characteristic qubit frequency to be equal 10 GHz, for simplicity). We propose the use of the magnetic field acting on the Josephson energy of the qubit for magnetization reversal which, in one sense, is equivalent to the precession of the magnetic moment in the *Y*-*Z* plane in the presence of a magnetic field along the *OX* axis (see Figure 1). The problem is consistent with the recent experimental works reporting the fast magnetization reversal induced by picosecond electromagnetic pulses in some specific magnetic media [25–27]. However, in our case, the problem is solved for a simple two-level system suitable for modeling the behavior of not only the superconducting flux qubits, but the atomic systems, including promising molecular magnetic memory cells, atom-like spins in semiconductors, magnetic cluster inclusion in a Josephson junction weak link, etc. [28–36].

This paper is organized as follows. The first subsection of the Results and Discussion describes the analytical approach for the analysis of two-level magnetic system dynamics. We provide this analysis for two-level systems with different magnetic moments and different energy splitting (superconducting flux qubit and atomic-based qubit) and two possible influences (oscillating magnetic field and unipolar field created by, e.g., the use of an SFQ pulse) as illustrated in Figure 1. Furthermore, in the next subsection, we use the expressions obtained for determining the parameters of external influence, ensuring optimal magnetic reversal. In the Conclusion, we also discuss the physical principles of implementation of the above mentioned operations with atomic-based elements for quantum information processing and superconducting quantum bits.

## Results and Discussion

### Model: two types of external impacts

The goal of our investigation is to provide the field-induced dynamics of a quantum magnetic system on the picosecond timescale, which is much faster than all decoherence processes taking place both in atomic-based cells and the superconducting qubits. Thus, the problem can be solved in the framework of the nonstationary Schroedinger equation (rather than the density matrix formalism):

[1]
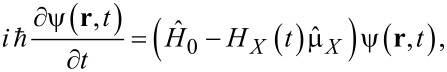


where 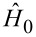 is the Hamiltonian of the unperturbed magnetic system, 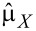 is the operator of the *X* projection of the magnetic dipole moment of the system and *H_X_*(*t*) is the external magnetic field that is considered in two different forms: as an oscillating or short but unipolar magnetic pulse. We consider a simple two-level magnetic quantum system where its state is described by the nonstationary wavefunction as:

[2]
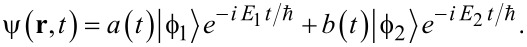


Here *E*_1_ and *E*_2_ are the energies of the stationary states 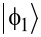 and 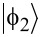, respectively, and the time-dependent probability amplitudes of the eigenstates *a*(*t*) and *b*(*t*) obey the condition |*a*(*t*)|^2^ + |*b*(*t*)|^2^ = 1. We suppose (see Figure 1) that at *t* = *t*_in_ an external magnetic field, **H**(*t*), directed along the *OX* axis, starts to act on the system and the spin-quantization axis coincides with the *OZ* direction and the initial condition is chosen in the form *a*(*t* = *t*_in_) = *a*_0_, b(*t* = *t*_in_) = *b*_0_.

The general equations for the probability amplitudes can be written in the form [37]:

[3]
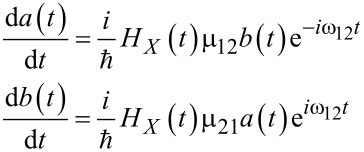


with 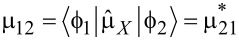 and 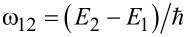.

The system dynamics is strongly determined by the type of external influence **H**(*t*) and evidently depends on the magnetic properties of the system, specifically on the value of μ_12_. In this work we consider two different limits of the examined quantum system, the atomic-based system and the macroscopic superconducting qubit with an extremely large characteristic magnetic moment. Thus, considering the external influences on the system, it is reasonable to focus on the two limiting cases.

We begin with the simplest case of interaction with an oscillating magnetic field in order to create a basis for the analysis of the magnetization reversal process or initialization:

[4]
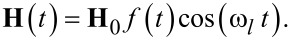


Under resonance conditions (ω*_l_* = ω_12_), and in the framework of the rotating wave approximation (that assumes in addition for a characteristic pulse duration, τ, the condition ω_12_τ >> 1), the solution of Equation 3 is well known and can be presented in the form:

[5]
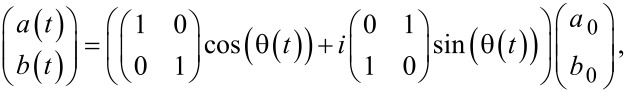


where 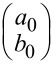 is the initial condition and

[6]
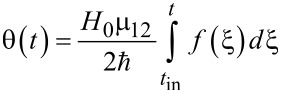


is proportional to the pulse area that determines the efficiency of the field-induced transitions. The lower limit in the integral corresponds to the initial time of the pulse action. Equation 5 allows for the calculation of the parameters of the external action needed to achieve a requested state of the quantum system at a certain time. However, we are interested in obtaining simple estimates of the duration of a basic operation: the magnetization reversal process transferring the system from the lower to the upper state (from *a*_0_ ≡ *a*(*t* = *t*_in_) = 1 to *a*(*t*_rev_) = 0).

For the Gaussian envelope, 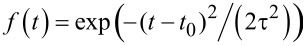, with characteristic duration τ (and *t*_in_ → −∞) we obtain the population of the lower (initial) state of the system, *W_a_*(*t*), given by:

[7]
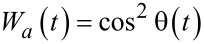


where

[8]
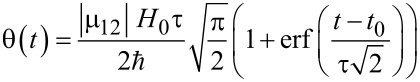


and


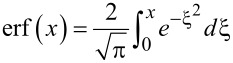


is the well-known error function, where in the general case, 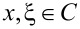. The analysis of this result is given below in the following subsection. It should be noticed that the obtained solution has a classical analogy in terms of correspondence between the trajectories of the introduced “pseudo-spin” vector and the classical Bloch vector discussed in [37].

We now move to the case of interaction with a short unipolar magnetic field. The investigation of the influence of a unipolar pulse (when ω*_l_* = 0) became critical in recent years in connection with the possibility to create magnetic field pulses of picosecond duration [16]. The time-dependent field is then given by:

[9]
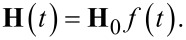


In matrix notation the problem looks quite simple:

[10]
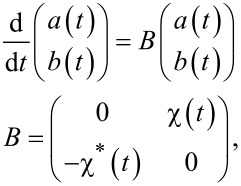


where 
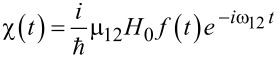
.

In the general case, this problem cannot be analytically solved due to the presence of the complex exponential function. We seek the approximate solution of Equation 10 in the form of the matrix exponential representation:

[11]
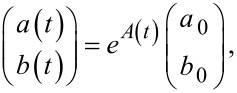


where the matrix exponent can be understood as a sum


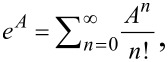


where the time-dependent matrix *A*(*t*) obeys the differential equation 
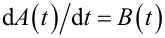
. The solution to this equation is given by:

[12]
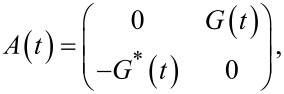


where


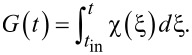


Introducing the notation *z*(*t*) = |*G*(*t*)|, the approximate Equation 11 can be written in the form:

[13]
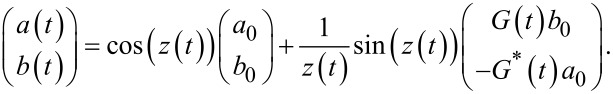


Equation 13 gives an exact solution of Equation 10 only if [*A*, d*A*/d*t*] = 0.

For the initial condition, *a*_0_ = 1, *b*_0_ = 0, we have the following expression for the population of the lower level:

[14]
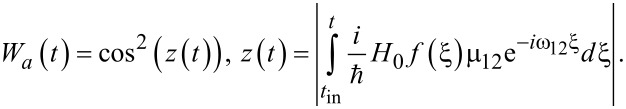


For the Gaussian envelope *f*(*t*) = exp(−(*t*−*t*_0_)^2^/(2τ^2^)) with characteristic duration τ

[15]
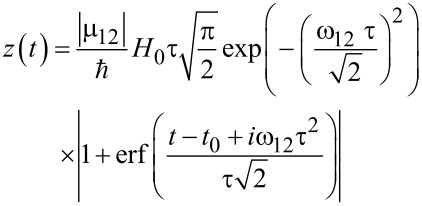


Formally, Equation 14 and Equation 15 seem to look very similar to Equation 7 and Equation 8. However, in this case, the repopulation of the qubit states is determined by the integral 
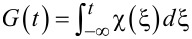
, which vanishes if the factor exp(−*i*ω_12_*t*) oscillates much faster than the function *f*(*t*) is changed, which can be easily seen from the exponentially decreasing term in Equation 15. However, it should be noted that in the case of superconducting flux qubits, the energy difference between the qubit levels corresponds to a ω_12_ in the GHz frequency range. For picosecond magnetic field pulses, this results in the condition ω_12_τ << 1. This condition provides the validity of our approximate solution and prevents the integral *G*(*t*) to tend towards zero, leading to the possibility of efficient transitions between the qubit levels.

Below the function *z*(*t*) that determines the solution for Equation 13 is found for two specific types of the nonadiabatic unipolar magnetic influence: the rectangular pulse (*t*_in_ = 0) and the RSFQ pulse (*t*_in_ → −∞). For the case of the rectangular envelope,

[16]
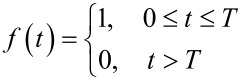


and

[17]
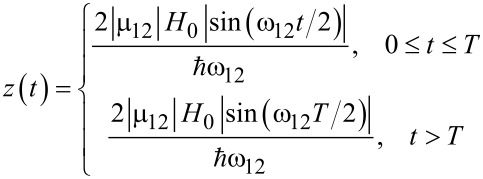


where in the limit of ω_12_τ << 1 (here τ ≡ *T*) gives rise to the usual Rabi oscillations with characteristic frequency of 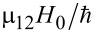. The reason for this lies in the nonadiabatic tendency of such a pulse, and therefore, its rather large spectral that which covers the energy shift between two levels.

The case of the fluxon pulse is of great importance due to the high possibility of the RSFQ-state management of superconducting qubits. The envelope is known to be of the following form [23–24]:

[18]
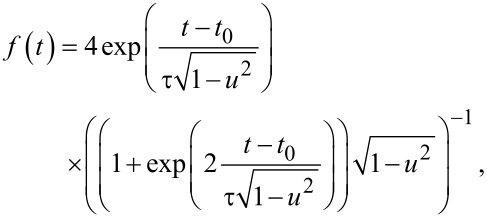


where *u* is the normalized velocity of the SFQ pulse (and *t*_in_ → −∞). For ω_12_τ << 1, the pulse area, which determines the time-dependent transitions in the system, is given by:

[19]
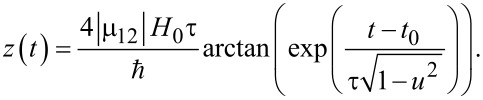


Notice that in this case the coherent control of the field-induced transitions can be provided by the variation of an additional parameter, the normalized pulse velocity, *u*.

### Magnetization reversal

We now analyze the results corresponding to the analytical solutions obtained in the previous section for oscillating quasi-resonance and unipolar magnetic pulses and their influence on the atomic and superconducting qubit systems. A dramatic difference between the atomic and superconducting qubits exists in the value of the matrix element of the magnetic dipole moment, which (for allowed transitions) appears to be 5 (or even more) orders of magnitude larger for the superconducting systems. Due to this fact, the magnetic transitions in superconducting systems can be even much more efficient than the allowed electric dipole transitions in atomic qubits.

**Figure 2:**
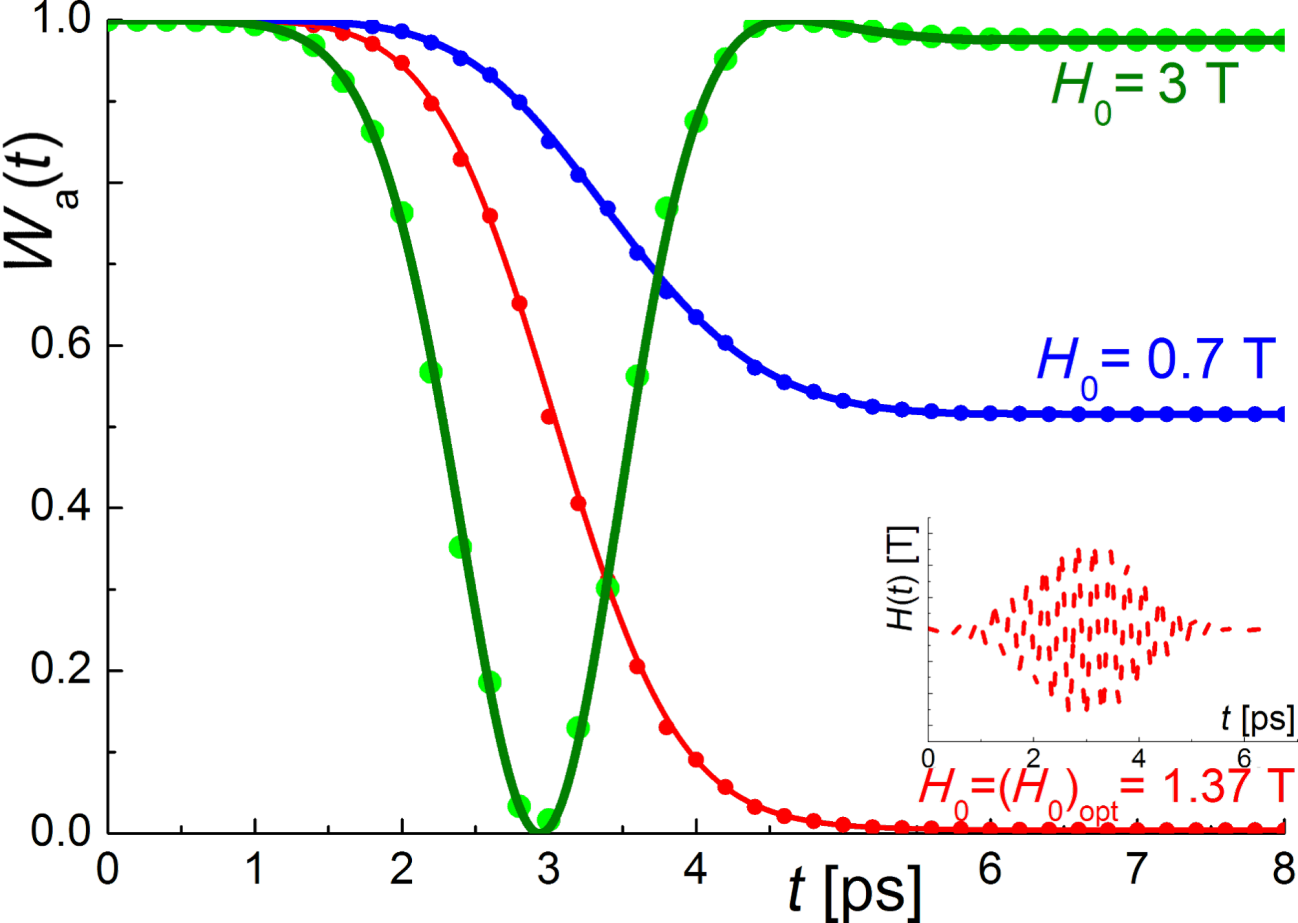
The dynamics of the ground level population, *W_a_*, of an atomic system (µ_12_ = 10µ_0_, ω_12_ ≈ 2·10^13^ Hz, *a*_0_ = 1, *b*_0_ = 0) for the case of an oscillating Gaussian magnetic field pulse (ω*_l_* = ω_12_, *t*_0_ = 3 ps, τ = 1 ps). The filled circles are the numerical calculations and the solid lines are the analytical approaches described using Equation 7 and Equation 8. The magnetic field acting on the system is presented in the inset (dashed lines).

**Figure 3:**
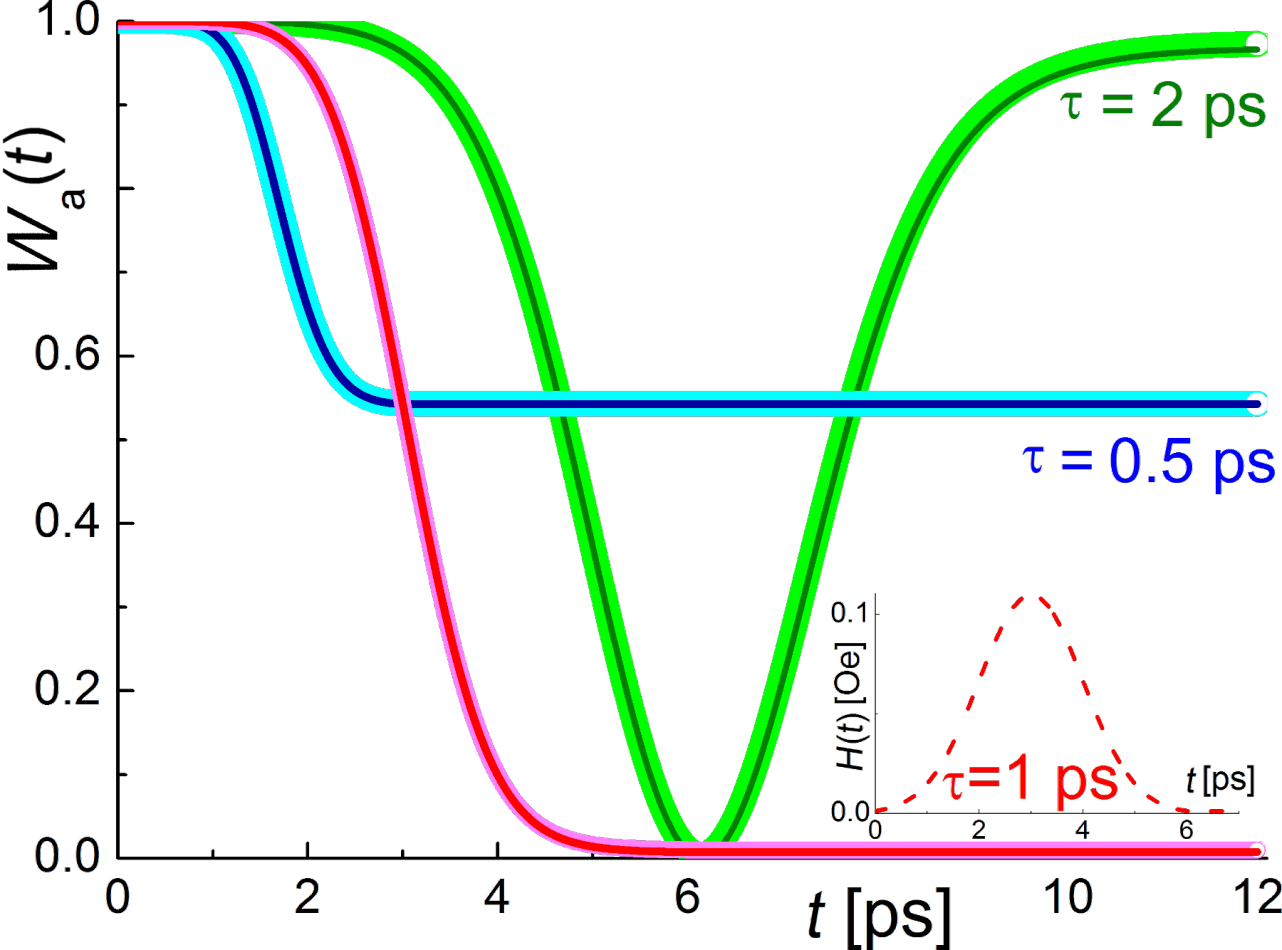
The dynamics of the ground level population, *W_a_*, of the flux qubit system (µ_12_ = 10^6^µ_0,_ ω_12_ ≈ 10^10^ Hz, *a*_0_ = 1, *b*_0_ = 0) for the case of a unipolar Gaussian magnetic field pulse (*H*_0_ ≈ 0.1 Oe, *t*_0_ = 3τ). The filled circles are the numerical calculations and the solid lines are the analytical approaches described using Equation 14 and Equation 15. The magnetic field acting on the system is presented in the inset (dashed lines).

In this work, we are mostly interested in the magnetization reversal process of two-level systems corresponding to the change in the probability amplitude of the lower state *a*(*t*), for example, from 1 to 0 during the influence of the external magnetic pulse. For the two cases considered, the oscillating and the unipolar field, the time for substantial change in the level population is determined by the area under the envelope of the magnetic field pulse (Equations 7 and 8, and Equations 14 and 15, respectively). If this area is too small, the magnetization reversal will not completely occur, and we can only achieve demagnetization (neither *a*(*t*) nor *b*(*t*) tend to zero at *t* → ∞). If this area is too large, partial magnetization reversal will occur, and the rest of the area is used for further change of level populations, also leading to demagnetization as clearly demonstrated in Figure 2 and Figure 3. These results are obtained for the atomic qubit under an oscillating pulse and the unipolar “Gaussian impact” in a superconducting flux qubit, respectively. The dependencies are very similar for both considered cases except the values of the characteristic magnetic field amplitude required to provide transitions on a picosecond timescale. This appears to be even less than 1 Oe in the case of the superconducting qubit because of the huge matrix element of magnetic momentum.

The performed numerical calculations based on the 4th order Runge–Kutta method demonstrate a perfect agreement with the obtained analytical solution that evidently proves the validity of the rotating wave and nonadiabatic pulse approximations, which are used in the cases of the oscillating and unipolar magnetic fields, respectively.

With regards to an optimum peak value of the magnetic field needed for the magnetization reversal, it can be easily found from the condition that the total pulse area (Equations 6, 7 and 14) should be equal to π/2, with the external magnetic pulse action reversing the system completely without further changes in its quantum state. In this optimal case, the magnetization reversal time, *t*_rev_, is equal to the effective duration of the external action, *T*, which in the case of a Gaussian envelope one can set as 6τ (corresponding to more than 99% of the area under the curve at *t*_0_ = 3τ). The optimal value of the peak magnetic field can be found from the following relations:

[20]
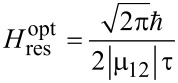


for the resonant oscillating Gaussian pulse,

[21]
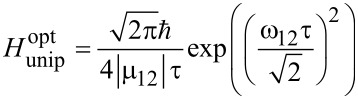


for a unipolar Gaussian pulse, and

[22]
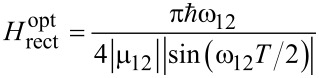


for a unipolar rectangular pulse.

Note that in the case of the unipolar influence (opposite to the case of the oscillating field), the effective reversal is possible only in the limit for which analytical expressions have been obtained: ω_12_τ << 1. The performed numerical calculations confirm that in the case of ω_12_τ ≥ 1, the exponentially growing factor in Equation 21 excludes the possibility of the process of interest under reasonable magnetic fields. A similar situation takes place in the case of the SFQ pulse, for which the optimal magnetic field strength can be achieved in the limit of ω_12_τ << 1 only and is given by:

[23]
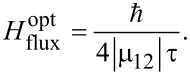


**Figure 4:**
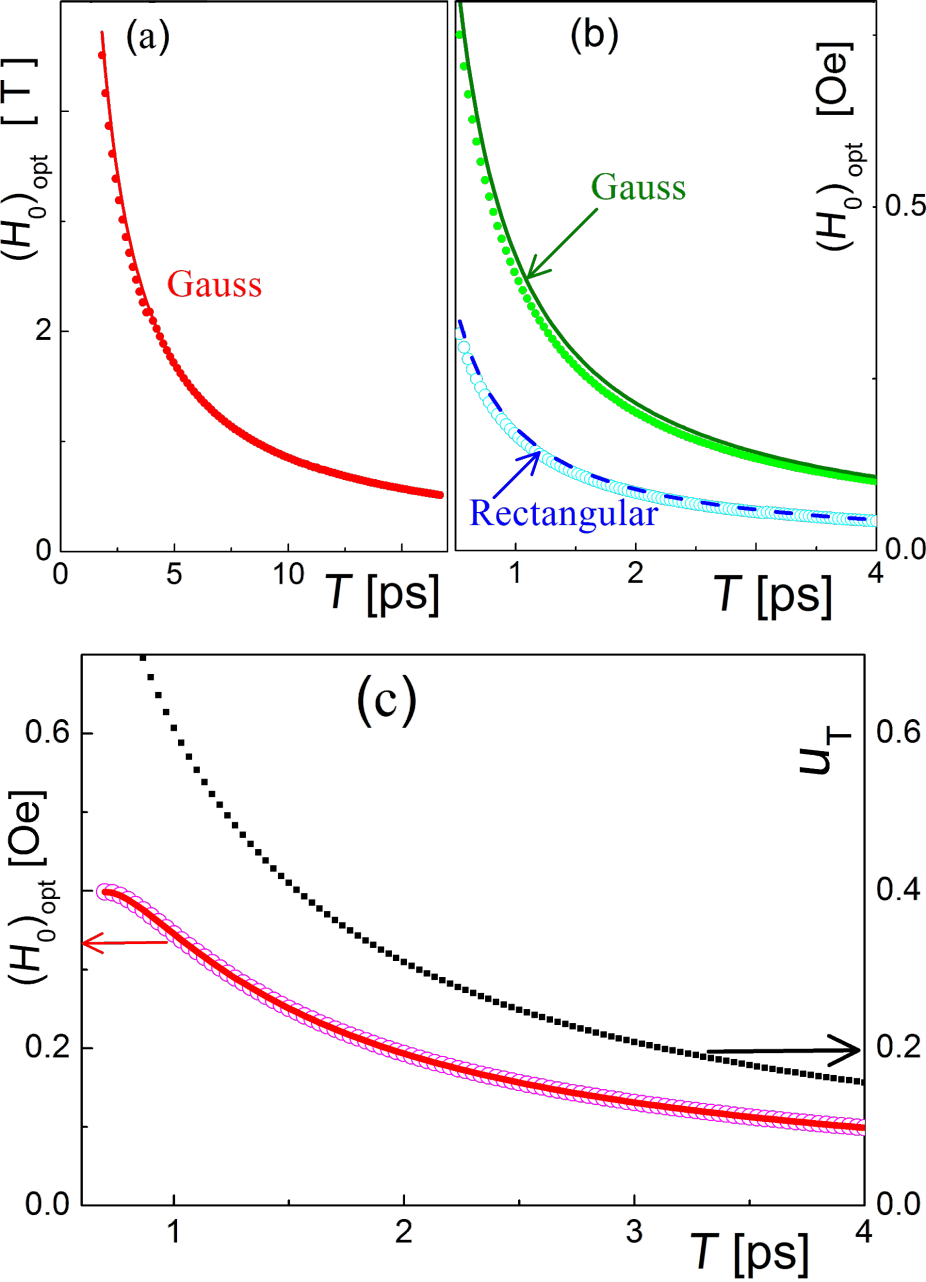
Dependence of the optimal magnetic field value versus the external magnetic pulse duration, T, for (a) the atomic-based qubit in an oscillating magnetic field (µ_12_ = 10µ_0_, ω*_l_* = ω_12_ ≈ 2·10^13^ Hz, *t*_0_ = 3τ, T = 6τ); (b) the superconducting flux qubit (µ_12_ = 10^6^µ_0,_ ω_12_ ≈ 10^10^ Hz) under the influence of unipolar impact with a Gaussian (ω*_l_* = 0, *t*_0_ = 3τ, T = 6τ) and rectangular (T = τ) envelope; (c) the superconducting qubit interacting with the passing fluxon. The filled circles are the numerical calculations and the solid lines are the analytical approaches described using Equations 20–23. The squares represent the fluxon speed providing appropriate duration of impact for the region of qubit–fluxon interaction of 0.5 μm.

Equations 21–23 (under the condition ω_12_τ << 1 for unipolar pulses) seem to be very similar and represent an expected inverse proportionality between the optimal magnetic field strength and the characteristic pulse duration. Figure 4 illustrates the dependence of the optimal magnetic field value for magnetization reversal versus the effective value for the external impact duration obtained for different pulses and different qubit types, both numerically and analytically (from Equations 20–23). The magnetic field strengths needed for magnetization reversal on a picosecond timescale can be easily seen from Figure 4.

From Figure 4a it can be seen that in the case of the oscillating resonant pulse, the magnetization reversal time is limited by the condition ω_12_τ >> 1 and corresponds to a timescale determined by the energy splitting between the eigenstates of the system. In the case of a unipolar impact, magnetization reversal is possible for times much shorter than 1/ω_12_; however, such a significantly nonadiabatic impact means that the envelope spectrum is broad enough to cover the eigenfrequencies of the atomic- or superconducting-qubit and may result in the population of additional nonresonant levels.

This simple rule – for the effective magnetization reversal a relatively large pulse duration is required for the oscillating field impact and a rather small one for the unipolar – is fully confirmed in the classical solution of the problem, manifesting the precession of the magnetic moment. The estimates obtained for two-level systems in the cases of unipolar (ω_12_τ << 1) and oscillating (ω_12_τ >> 1) impacts for the dynamics of the projection of the magnetic moment on the *Z*-axis, 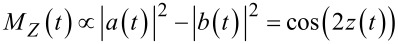 or cos(2θ(*t*)), respectively, coincide given the specified conditions with the predictions for the “magnetic management” in magnetic memory cells on a picosecond timescale based on the use of the Landau–Lifshitz–Gilbert equation [38–40]. Indeed, in a well-known classical macro-spin approximation, one can arrive at the formula for the component of magnetization dynamics, **M**, in a form equivalent to that obtained above for a quantum system. The Landau–Lifshitz–Gilbert equation can be written as follows:

[24]
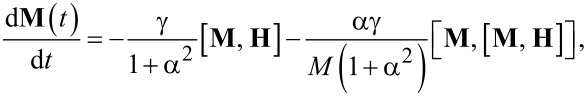


where γ is the gyromagnetic ratio, **M**(*t* = *t*_in_) = {0, 0, *M*}, and **M**(*t* = *t*_rev_) = {0, 0, −*M*}. The magnetic field is given in the form **H**(*t*) = **n***_X_H*(*t*) + **n***_Z_H_Z_*, where *H_Z_* > 0 is a constant field; the presence of which allows for the definition of the so-called Larmor frequency, Ω_L_ = γ*H_Z_*. Classical damping can be set as α = 0 for the simplest case when the decoherence processes in the quantum model can be neglected.

For *H*(*t*) = *H*_0_*f*(*t*)cos(ω*_l_t*), with ω*_l_* = Ω_L_, Ω_L_τ >> 1 one can arrive at:

[25]
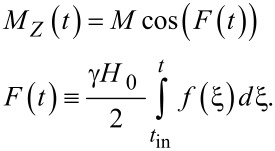


For the Gaussian envelope,

[26]
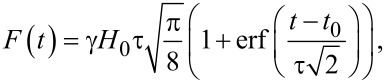


which (for a gyromagnetic ratio of 
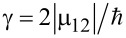
) fully agrees with the expression 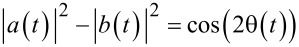 (see Equation 8) and leads to a very simple expression for the magnetization reversal timescale:

[27]
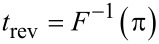


It is now obvious that for the classical problem, Ω_L_ is equivalent to ω_12_ for the quantum one.

For a unipolar field, *H*(*t*) = *H*_0_*f*(*t*) with the extra condition that Ω_L_τ << 1 and one can arrive at an analogous expression:

[28]
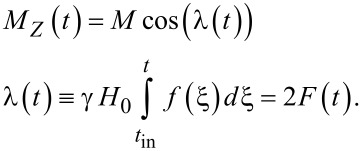


This formula corresponds to 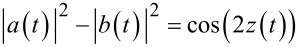 from Equation 14 and Equation 15 for the quantum case if Ω_L_ = ω_12_ and 
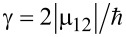
, and leads to an analogous expression for the magnetization reversal timescale:

[29]
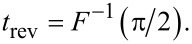


**Figure 5:**
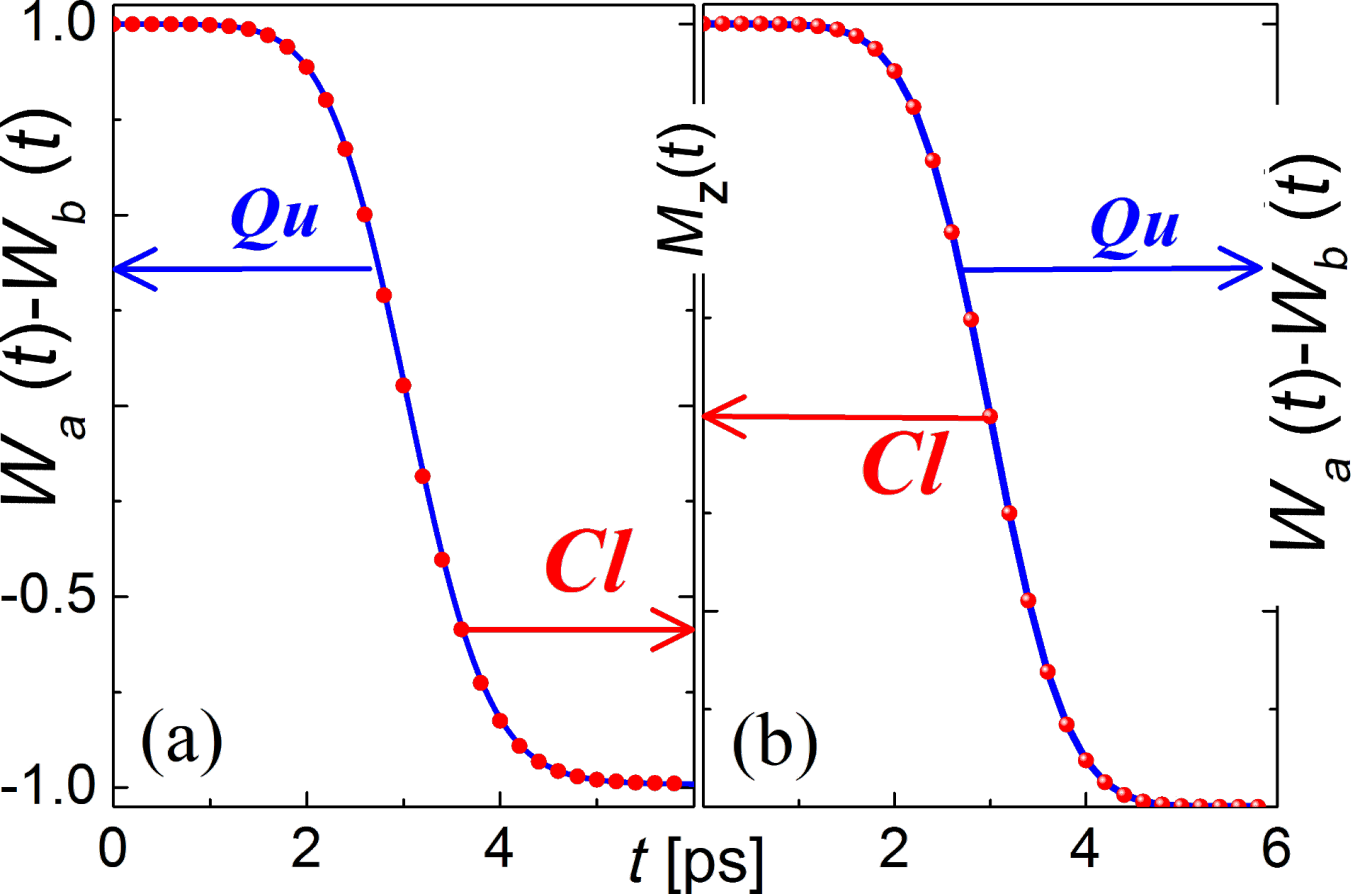
The ground level population dynamics of both the atomic-based qubit for an oscillating magnetic field impact (a): µ_12_ = 10µ_0_, ω*_l_* = ω_12_ = Ω_L_ ≈ 2·10^13^ Hz, *H*_0_ ≈ 1.37 T, τ = 1 ps) and the flux qubit system for a unipolar magnetic field impact (b): µ_12_ = 10^6^µ_0_, ω*_l_* = 0; ω_12_ = Ω_L_ ≈ 10^10^ Hz, *H*_0_ ≈ 0.1 Oe, τ = 1 ps) in comparison with the predictions of the Landau–Lifshitz–Gilbert equation (
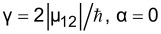
) for the *Z*-projection of the magnetization behavior. The filled circles are classical calculations and the solid lines are the results for the quantum model.

Figure 5 illustrates the similarity between the dynamics of the process of magnetization reversal within the classical and quantum approaches with the oscillating magnetic field impact (when ω_12_τ >> 1*,* Ω_L_τ >> 1, and Ω_L_ = γ*H*_0_), and in the case of short unipolar pulses (under the condition ω_12_τ << 1*,* Ω_L_τ << 1). Note that here in the intermediate case, Ω_L_τ ≈ 1, the effective reversal is also impossible because of the alternating sign in the *M_Y_*(*t*)·*H_X_*(*t*) term in Equation 24. According to the simple precession equation, this sign defines the sign of d*M_Z_*/d*t*: only divergent impacts on a timescale comparable with the pulse duration can lead to demagnetization in the system, which is confirmed by the results of the numerical simulations.

It is important to pay attention to this profound analogy between the quantum and the classical results in order to separate the macroscopic and quantum modes in mesoscopic systems, which is often a difficult task [41–42]. Moreover, it seems promising to simulate the behavior of magnetic metamaterials based on artificial Josephson atoms in sufficiently strong fields [43] using standard classic software designed to study the magnetization reversal processes in large magnetic clusters [44].

### The three-level system and the Λ-scheme

**Figure 6:**
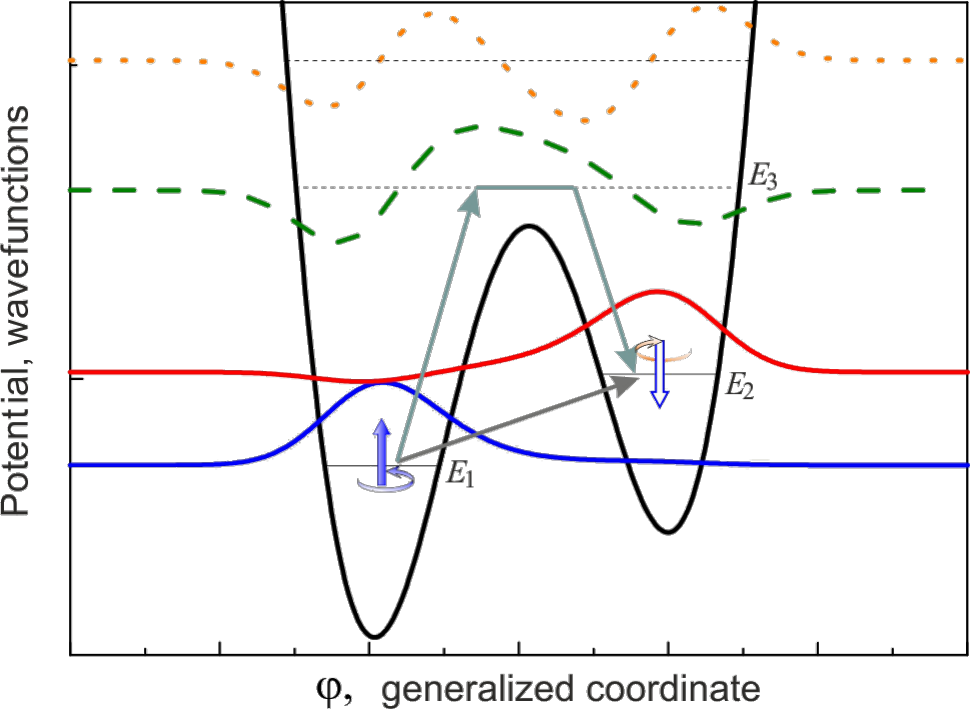
The potential energy and the wavefunctions for the flux-driven, three-junction qubit (described in [2]), where the Josephson energies of the elements are *E*_J_, *E*_J_ and 0.9*E*_J_; the Josephson energy scale, *E*_J_, is 80 times larger than the characteristic Coulomb energy of the elements.

As was previously mentioned, both the resonant and unipolar pulses either give rise to some limitations on the magnetization reversal time or populate additional nonresonant levels. However, it is possible for a superconducting flux qubit to avoid the main disadvantage of the unipolar influence under certain conditions, that is, to not excite the system on undesirable levels. The typical potential energy and the eigenfunctions for the flux-driven, three-junction qubit are presented in Figure 6. The selective excitation of magnetization reversal is possible if the parameters of the qubit together with the parameters of the unipolar impact ensure compliance with the condition ω_12_τ << 1. This is respect to transitions between states with well-defined values of the magnetic moment of the “0” and “1” states (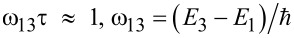, etc.).

**Figure 7:**
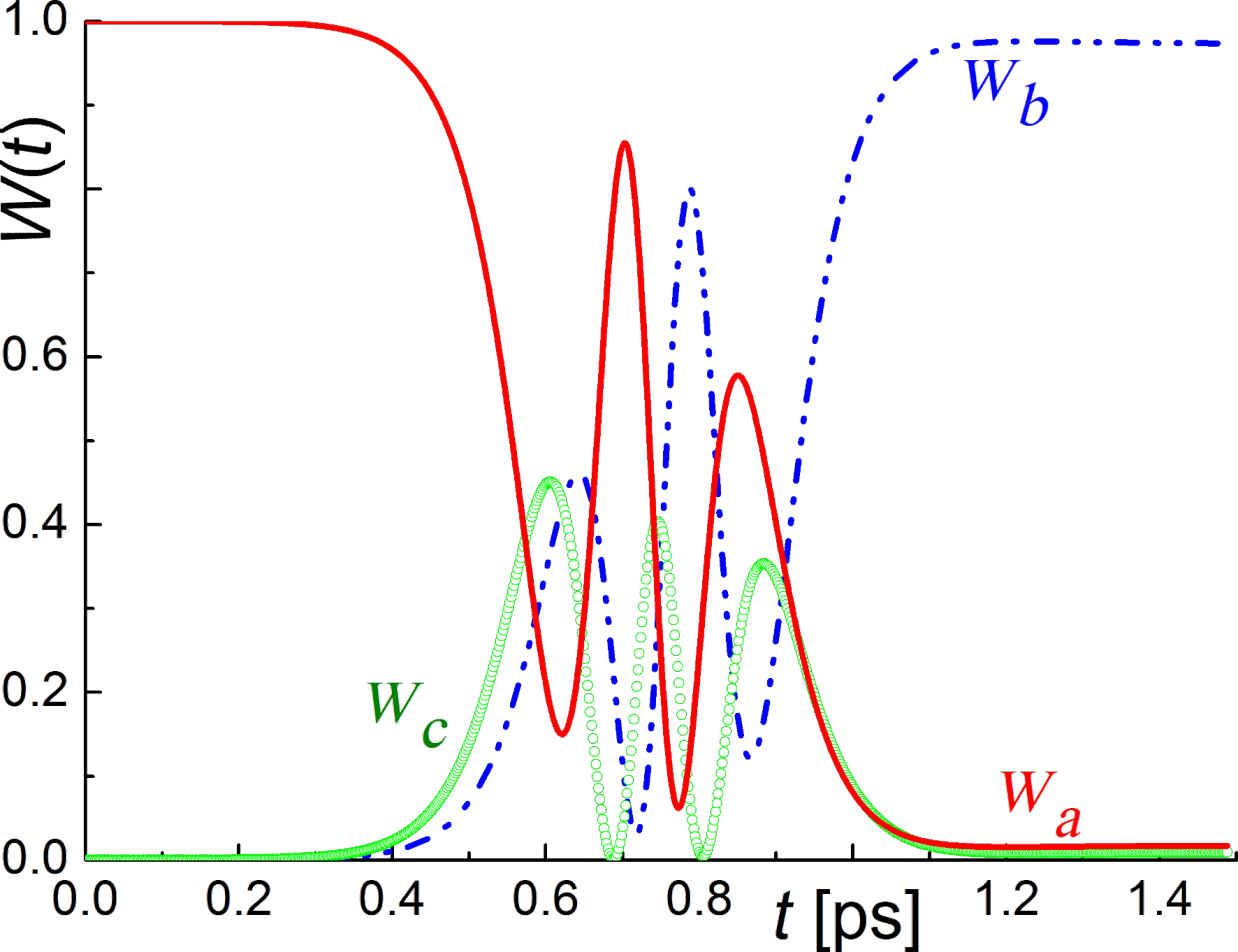
The dynamics of the ground level population, *W_a_*, and the populations of the two lowest excited levels, *W_b_* and *W_c_*, of the flux qubit system (µ_12_ = 10^6^µ_0_, 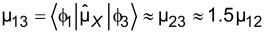, ω_12_ ≈ 10^10^ Hz, ω_13_ ≈ 3ω_12_, *a*_0_ = 1, *b*_0_ = *c*_0_ = 0) for the superconducting qubit interacting with the passing fluxon (duration of external impact is about 1 ps).

The theoretical possibility of such selective magnetization reversal of a superconducting flux qubit using an SFQ pulse is confirmed by the results of the numerical simulation of the three-level system dynamics (see Figure 7).

An alternative, promising procedure is based on the so-called Λ-scheme, involving not only the two lowest energy levels, but also the excited one. For such three-level systems, the third highly excited state, 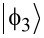, is the superposition of the basis wavefunctions with well-defined values of the magnetic moment (see Figure 6), so the transitions from each of the two lower states are allowed. The dynamics of the system under the magnetic pulse action can be investigated by analogy with Equations 1–3.

[30]
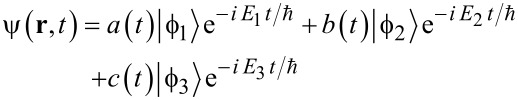


For the simplest case, when *H_X_*(*t*) = *H*_0_cos(ω*_l_t*), 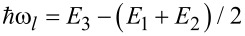 we have:

[31]
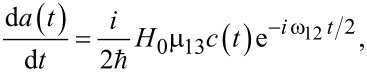


[32]
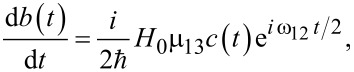


[33]
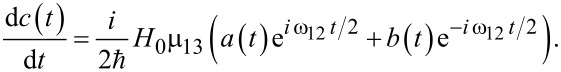


Here we assume for simplicity that 
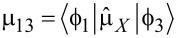
 
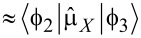
, and solving the matrix Equations 31–33 for the initial condition *a*_0_ ≡ *a*(*t* = 0) = 1, one can obtain for the amplitude of the final state with the “inverted” magnetic moment:

[34]
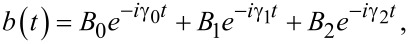


[35]
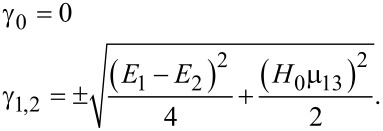


Hence, the population of the excited basis level, *W_b_*, oscillates as:

[36]
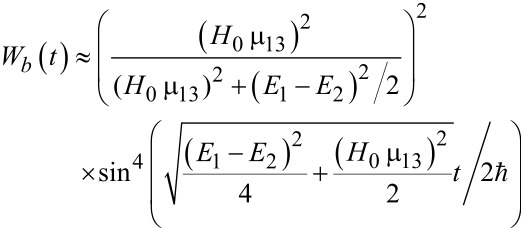


with a characteristic time of about 
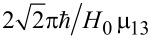
. It should be emphasized that for the considered Λ-scheme, the characteristic time of magnetization reversal is limited by the value (no shorter than) 1/ω_13_, rather than the value 1/ω_12_ as for the direct resonant Rabi transitions 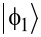 and 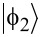. Therefore, this characteristic time is much shorter since the resonant frequency ω_13_ appears to be several times larger than the energy gap between closely situated levels 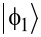 and 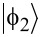. For this reason, the reversal time can be made much shorter than for the two-level system simply by increasing the field strength.

As one can see, the use of the alternative pathway allows for the increase in the speed of the transition between the basic magnetic states under the influence of multimode excitation, and experimental confirmation of this assumption is of particular interest both for the applications and for the fundamental studies in the field of quantum optics on a chip.

## Conclusion

In summary, one can conclude that for an atomic-based magnetic qubit, an oscillating magnetic field of about 1 T is required in order to achieve a magnetization reversal time of less than 0.01 ns. However, from a technological perspective, such magnetic field intensities are very difficult to achieve.

The strong coupling regime between a superconducting flux qubit and a resonator composed of inductances and capacitances was reported. Therefore, it was easy to put the impact of the oscillating field on artificial atom into practice (similar to the case studied in detail for usual atomic systems) [45–47]. At the same time, for a typical flux qubit (with ω_12_ ≈ 10 GHz) it is practically impossible to realize the effective magnetization reversal by the oscillating magnetic field pulse on a picosecond time scale. In this case, the oscillation period is comparable to the duration of the pulse and de facto we are dealing with an ineffective unipolar influence.

However, for the superconducting flux qubit, one needs the unipolar magnetic field pulse with an amplitude of about 1 Oe for the solution of the magnetization reversal problem on the picosecond timescale. Note that in this case the characteristic decoherence time is up to 10 μs [48].

It is possible to implement such an impact in the experiment due to the magnetic coupling of the superconducting quantum bit with a Josephson transmission line. During the propagation of the fast single flux quantum pulse (fluxon) in the transmission line, the magnetic field interacting with the qubit can be represented exactly as a unipolar pulse. The shape of this pulse can be calculated as a convolution of the according function, representing the coupling between the qubit and magnetic flux of the vortex in a Josephson transmission line. The shape can be governed in situ with the relation between the driving force and losses in the system [24]. The speed of the fluxons required for the effective magnetization reversal of superconducting flux qubits (see Figure 4c) is achievable for Josephson transmission lines that are designed and built using the latest advances in superconductor technology.

Moreover, the unusual ability to create the so-called Δ-type artificial atom (forbidden by the selection rules for typical atoms) on the basis of flux-driven three-junction loops with broken Hamiltonian symmetry allows us to consider magnetization reversal within the Λ-scheme (along with the usual magnetization reversal) [49–51]. The latter option seems to be currently the best way for simple operations with the quantum states on a subnanosecond timescale.
